# Current Clinical Practice of Laboratory Testing of the Hemostasis and Coagulation System in Patients with Sepsis: A Nationwide Observational Study in Japan

**DOI:** 10.31662/jmaj.2023-0151

**Published:** 2024-02-05

**Authors:** Kazuma Yamakawa, Hiroyuki Ohbe, Ryo Hisamune, Noritaka Ushio, Hiroki Matsui, Kiyohide Fushimi, Hideo Yasunaga

**Affiliations:** 1 Department of Emergency and Critical Care Medicine, Osaka Medical and Pharmaceutical University, Takatsuki, Japan; 2 Department of Clinical Epidemiology and Health Economics, School of Public Health, The University of Tokyo, Tokyo, Japan; 3 Department of Emergency and Critical Care Medicine, Tohoku University Hospital, Sendai, Japan; 4 Department of Health Policy and Informatics, Tokyo Medical and Dental University Graduate School of Medicine, Tokyo, Japan

**Keywords:** disseminated intravascular coagulation, molecular marker, prothrombin fragment 1 + 2, soluble fibrin, thrombin-antithrombin complex

## Abstract

**Introduction:**

The clinical benefit of hemostasis molecular indicators such as thrombin-antithrombin complex (TAT), soluble fibrin (SF), and prothrombin fragment 1 + 2 (F1+2) for the diagnosis of disseminated intravascular coagulation (DIC) is reported. Recently, novel DIC diagnostic criteria that adopt them were proposed in Japan. Despite the theoretical understanding of their function, the practical use of these markers remains unclear. The present study aimed to provide a descriptive overview of current clinical practice regarding the measurement of hemostasis markers in sepsis management in Japan.

**Methods:**

This retrospective observational analysis used the Japanese Diagnosis Procedure Combination inpatient database containing data from more than 1500 acute-care hospitals in Japan. We identified adult patients hospitalized for sepsis between April 2018 and March 2021. Descriptive statistics for measuring several hemostasis laboratory markers were summarized using patient disease characteristics, hospital characteristic, and geographical location.

**Results:**

This study included 153,474 adult sepsis patients. Crude in-hospital mortality was 30.0%. Frequency of measurement of fibrinogen, fibrin degradation products (FDP), and D-dimer in sepsis patients on admission was 43.2%, 36.1%, and 46.4%, respectively. Novel and specific hemostasis molecular markers such as TAT, SF, and F1+2 were seldom measured (1.9%, 1.7%, and 0.02%, respectively). Hemostasis molecular markers were more frequently measured with progression of thrombocytopenia. Measurement of these clinically favorite hemostasis markers was influenced not only by disease characteristics but also hospital characteristic or geographical location.

**Conclusions:**

Hemostasis molecular markers such as TAT, SF, and F1+2 were rarely measured in clinical settings. Although adopted by several DIC scoring systems, neither fibrinogen, FDP, nor D-dimer was routinely measured.

## Introduction

Sepsis, a severe response to infection causing a life-threatening state of organ failure, remains a major global health issue ^(1)^, with an estimated 48.9 million sepsis cases and 11 million sepsis-related deaths worldwide annually ^(2)^. Disseminated intravascular coagulation (DIC), a severe condition characterized by the formation of numerous small blood clots, disrupts blood flow, leading to severe organ damage and potentially fatal outcomes ^(3), (4), (5)^. Numerous studies have demonstrated that the mortality of sepsis patients with DIC was nearly twofold higher than that of sepsis patients without DIC ^(6), (7), (8)^. Therefore, for the management of sepsis, it is necessary to understand the pathophysiological mechanism and best management of DIC. To this end, the evaluation of several biomarkers of the hemostasis and coagulation system serves as an essential tool in both diagnosing and tracking DIC.

Diagnostic criteria for DIC commonly include measurements of these hemostasis and coagulation system markers such as platelet counts, fibrin degradation products (FDP), or D-dimer, prothrombin time, and fibrinogen ^(9), (10), (11)^. These indicators provide important insights about the patient’s coagulation status, and their trend can indicate a development toward DIC. Consequently, the utilization of these markers is an integral part of sepsis management as they guide clinicians in making informed decisions regarding patient care. However, despite the theoretical understanding of the function of these markers, their practical application remains unclear.

Recently, the Japan Society on Thrombosis and Hemostasis (JSTH) introduced new diagnostic criteria for DIC ^(12)^, which incorporate multiple molecular markers such as antithrombin (AT), thrombin-antithrombin complex (TAT), soluble fibrin (SF), and prothrombin fragment 1 + 2 (F1+2). Compared to prior diagnostic criteria for DIC, the JSTH criteria require a thorough evaluation of hemostasis and coagulation markers, but it is unknown how often these tests are performed in clinical practice. To address these knowledge gaps, we gathered data from the Diagnosis Procedure Combination (DPC) database, a nationwide administrative database in Japan. The present study aimed to give a descriptive review of the current clinical practice regarding the measurement of hemostasis and coagulation markers in sepsis treatment in Japan.

## Materials and Methods

### Design and setting

This was a retrospective observational study using routinely collected data. The study was approved by the Institutional Review Board of the University of Tokyo [approval number: 3501-(5) (May 19, 2021)]. The board waived the requirement for informed consent because of the anonymous nature of the data and because no information on individual patients, hospitals, or treating physicians was obtained.

We used the Japanese Diagnosis Procedure Combination inpatient administrative database, which contains discharge abstracts and administrative claims data for more than 1500 acute-care hospitals and covers approximately 90% of all tertiary-care emergency hospitals in Japan ^(13)^. The database includes the following patient-level data for all hospitalizations: demographic characteristics; diagnoses recorded with International Classification of Diseases, Tenth Revision (ICD-10) codes; daily procedures recorded using Japanese procedure codes; daily drug administrations; and admission and discharge status. A prior validation analysis of this database showed high specificity and moderate sensitivity for recorded diagnoses and high specificity and sensitivity for recorded procedures ^(14)^. The Sequential Organ Failure Assessment (SOFA) scores on the first day of sepsis treatment have been required to be input into the database for patients aged 15 and older with sepsis since April 1, 2018, in Japan. The database recorded the SOFA score as the lowest overall score on the first day of sepsis treatment.

### Study population

We identified all adult patients aged 15 years and older who were hospitalized for sepsis with a SOFA score recorded on the day of admission between April 1, 2018, and March 31, 2021. We excluded patients whose total SOFA scores were less than two on the day of admission according to the Sepsis-3 definition ^(15)^ and patients who had at least one incomplete score for each of the six organ systems used to generate the total SOFA score on the day of admission. All patients were followed up until they died or were discharged from the hospital.

### Data collection

We collected the following data on baseline patient characteristics: age, sex, body mass index, Charlson comorbidity index ^(16)^, total SOFA score on the day of admission, organ dysfunction on the day of admission, source of infection, arrived via emergency medical services, admission site (intensive care unit, high-dependency care unit, and general ward) on the day of admission, surgery under general anesthesia on the day of admission, hospital category (teaching hospital and tertiary emergency hospital), and annual hospital volume of sepsis. Organ dysfunction was determined if the SOFA subcomponent score was 2 or more. To determine the source of infection, we utilized the definition of ICD-10 codes reported previously (Supplementary Table 1) ^(17)^. We also collected in-hospital mortality as a general outcome.

As for the main focus of this study, we gather data on the following hemostasis laboratory markers within 7 days of admission: platelet count, prothrombin time (PT), activated partial thromboplastin time (aPTT), fibrinogen, FDP, D-dimer, AT, TAT, SF, F1+2, and plasmin-α2 plasmin inhibitor complex (PIC).

### Statistical analysis

The percentages of measurement of several hemostasis laboratory markers are summarized in both patient and hospital groups. Categorical variables are presented as numbers and percentages. Continuous variables are presented as mean and standard deviation (SD). Significance was determined by Pearson’s chi-squared test. All analyses were performed using STATA/SE 17.0 software (StataCorp, College Station, TX, USA).

## Results

**Figure 1.**
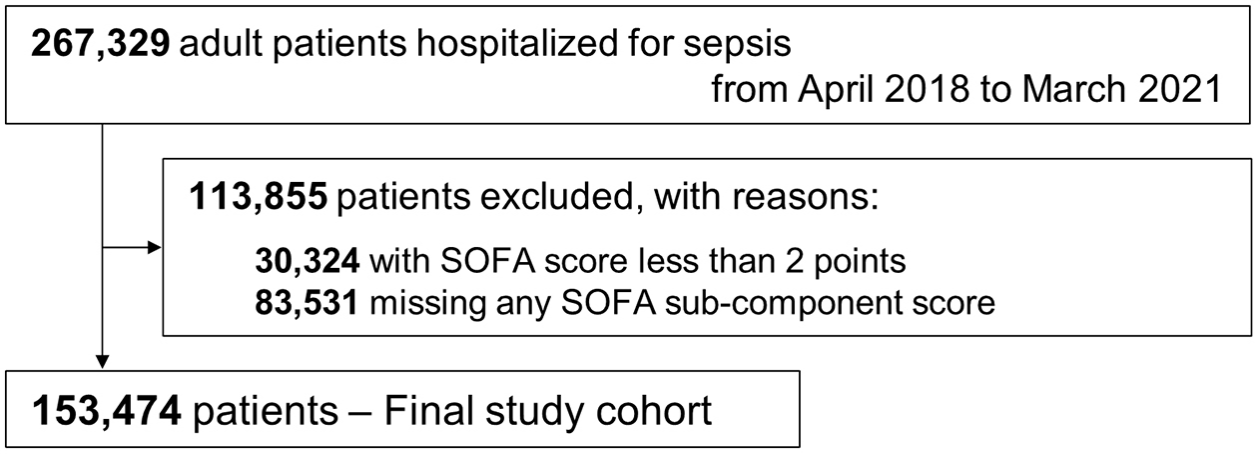
Patient flow diagram. SOFA, Sequential Organ Failure Assessment.

Following application of the inclusion and exclusion criteria, 153,474 adult sepsis patients were included in the current study (Figure 1). Baseline patient characteristics are shown in Table 1. Among these patients, the percentage of men was 56.6%, and the mean age was 76.0 years (SD, 13.4 years). The mean total SOFA score on the day of admission was 6.8 (SD, 3.9). The main admission site on the day of admission was a general ward in 58.5%, followed by high-dependency unit in 20.0% and intensive care unit in 21.5%. The source of infection was mainly the lung, abdomen, and urinary tract. The crude in-hospital mortality was 30.0%.

**Table 1.** Patient Characteristics.

Characteristic	Overall sepsis patients (n = 153,474)	
Age, year, mean (SD)	76.0	(13.4)
Male sex, n (%)	86,888	(56.6)
Body mass index, kg/m^2^, n (%)
<18.5	32,758	(21.3)
18.5-24.9	77,519	(50.5)
25.0-29.9	21,217	(13.8)
≥30.0	6,045	(3.9)
Missing	15,935	(10.4)
Charlson comorbidity index, mean (SD)	1.5	(1.7)
Total SOFA score, mean (SD)	6.8	(3.9)
Organ dysfunction, n (%)
Respiration	61,809	(40.3)
Coagulation	54,460	(35.5)
Liver	27,938	(18.2)
Cardiovascular	49,789	(32.4)
CNS	58,467	(38.1)
Renal	50,350	(32.8)
Source of infection, n (%)
Lung	32,733	(21.3)
Abdomen	29,682	(19.3)
Urinary tract	30,678	(20.0)
CNS	1,275	(0.8)
Skin/soft tissue	4,732	(3.1)
Cardiovascular	4,314	(2.8)
Other	61,432	(40.0)
Arrived via EMS, n (%)	91,769	(59.8)
Admission site, n (%)
General ward	89,800	(58.5)
HDU	30,672	(20.0)
ICU	33,002	(21.5)
Surgery with general anesthesia, n (%)	17,047	(11.1)
Hospital category, n (%)
Teaching	145,746	(95.0)
Tertiary	70,867	(46.2)
Annual hospital volume, patients/year, mean (SD)	326.7	(261.7)
In-hospital mortality, n (%)	46,035	(30.0)

Abbreviations: SD, standard deviation; SOFA, Sequential Organ Failure Assessment; CNS, central nervous system; EMS, emergency medical services; HDU, high-dependency unit; ICU, intensive care unit

**Figure 2.**
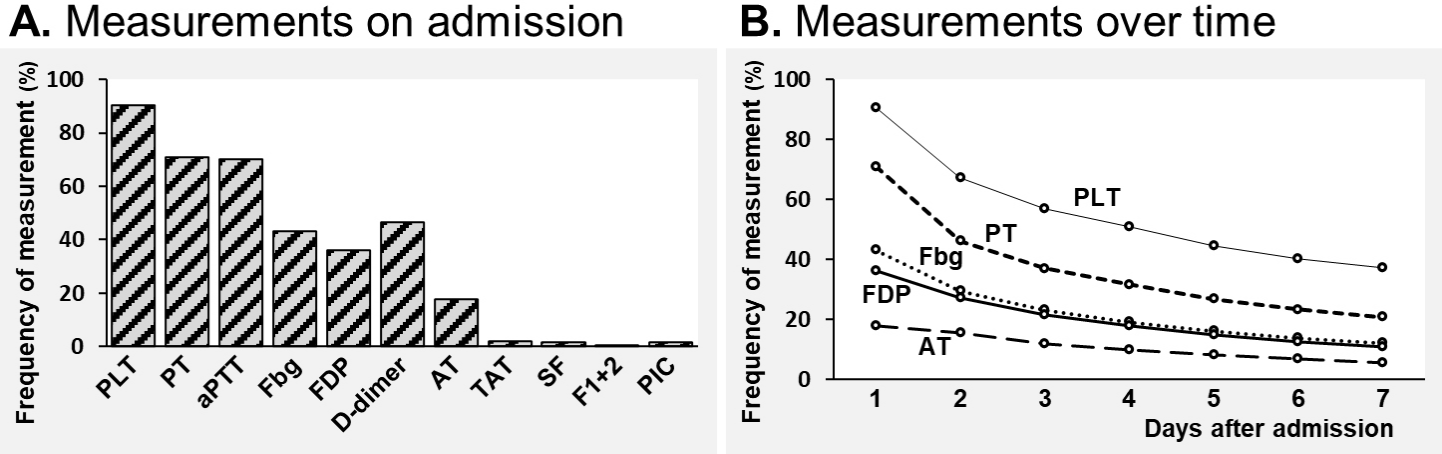
Frequency of measurement of hemostasis markers in overall sepsis patients. (A) Frequency of measurement on admission day. (B) Frequency of measurement over time during the first 7 days after admission. PLT, platelet count; PT, prothrombin time; aPTT, activated partial thromboplastin time; Fbg, fibrinogen; FDP, fibrin degradation products; AT, antithrombin; TAT, thrombin-antithrombin complex; SF, soluble fibrin; F1+2, prothrombin fragment 1 + 2; PIC, plasmin-α2 plasmin inhibitor complex.

The frequencies of measuring hemostasis markers in the overall sepsis patients are shown in Figure 2. The existing hemostasis markers such as fibrinogen, FDP, and D-dimer were measured in only about one-third to one-half of the sepsis patients on admission (43.2%, 36.1%, and 46.4%, respectively). The novel and specific hemostasis molecular markers such as TAT, SF, and F1+2 were seldom measured (1.9%, 1.7%, and 0.02%, respectively) (Figure 2A). The frequency of assessing these hemostasis markers gradually dropped as the hospital days passed (Figure 2B).

**Figure 3.**
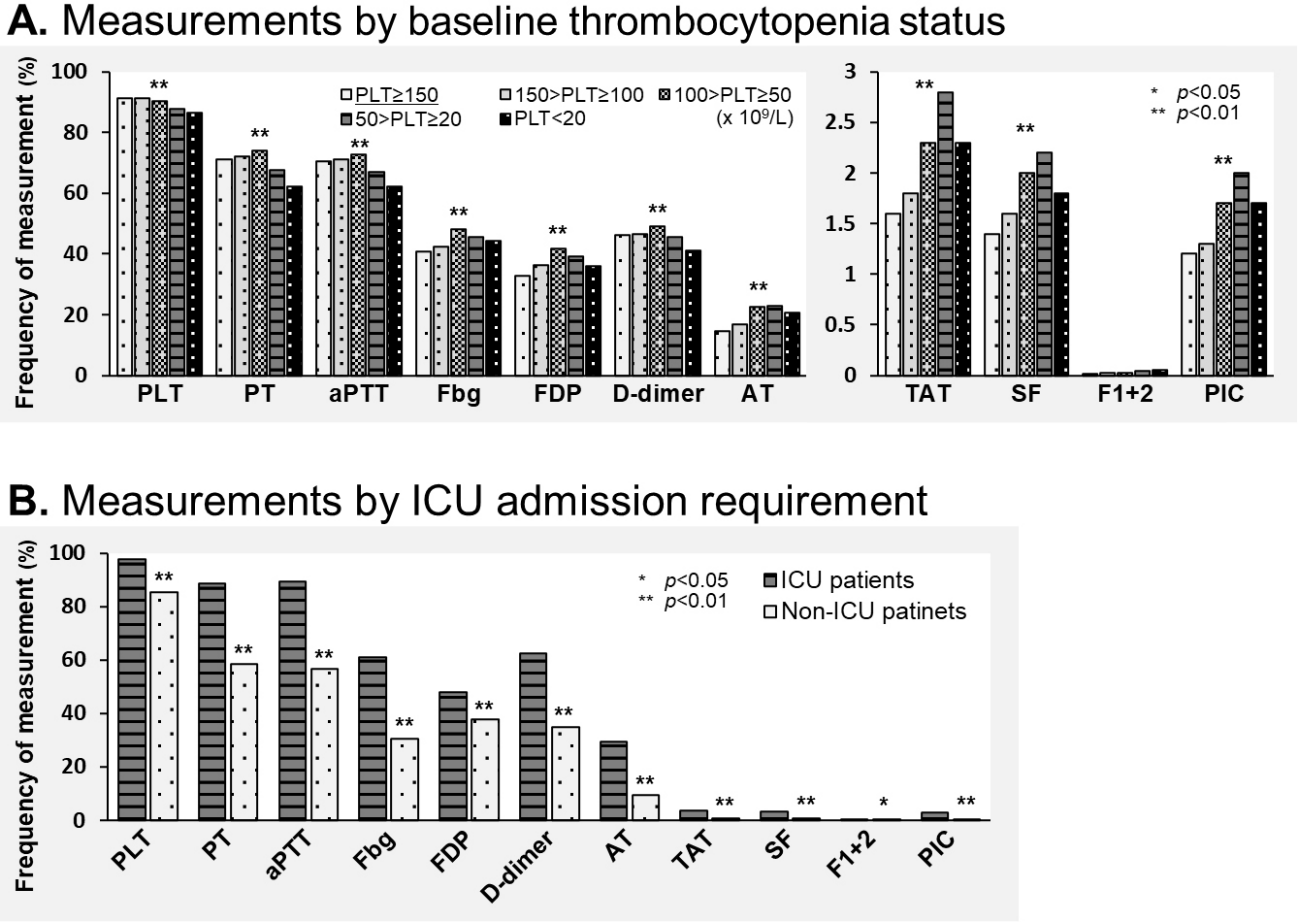
Measurement disparities according to patient status. (A) Frequency of measurement of hemostasis markers by thrombocytopenic status on admission. (B) Frequency of measurement of hemostasis markers by requirement of ICU admission. Significance was determined by Pearson’s chi-squared test. PLT, platelet count; PT, prothrombin time; aPTT, activated partial thromboplastin time; Fbg, fibrinogen; FDP, fibrin degradation products; AT, antithrombin; TAT, thrombin-antithrombin complex; SF, soluble fibrin; F1+2, prothrombin fragment 1 + 2; PIC, plasmin-α2 plasmin inhibitor complex; ICU, intensive care unit.

Measurement disparities according to patient status are shown in Figure 3. Global laboratory markers (such as platelet count, PT, aPTT, fibrinogen, FDP, and D-dimer) did not differ between the groups by baseline platelet count. However, specific hemostasis molecular markers (such as TAT, SF, and PIC) were more frequently assessed with progression of thrombocytopenia in the sepsis patients (Figure 3A). Both global laboratory markers and specific hemostasis molecular markers indicative of the patients’ disease severity were more frequently measured in patients in the ICU (Figure 3B).

**Figure 4.**
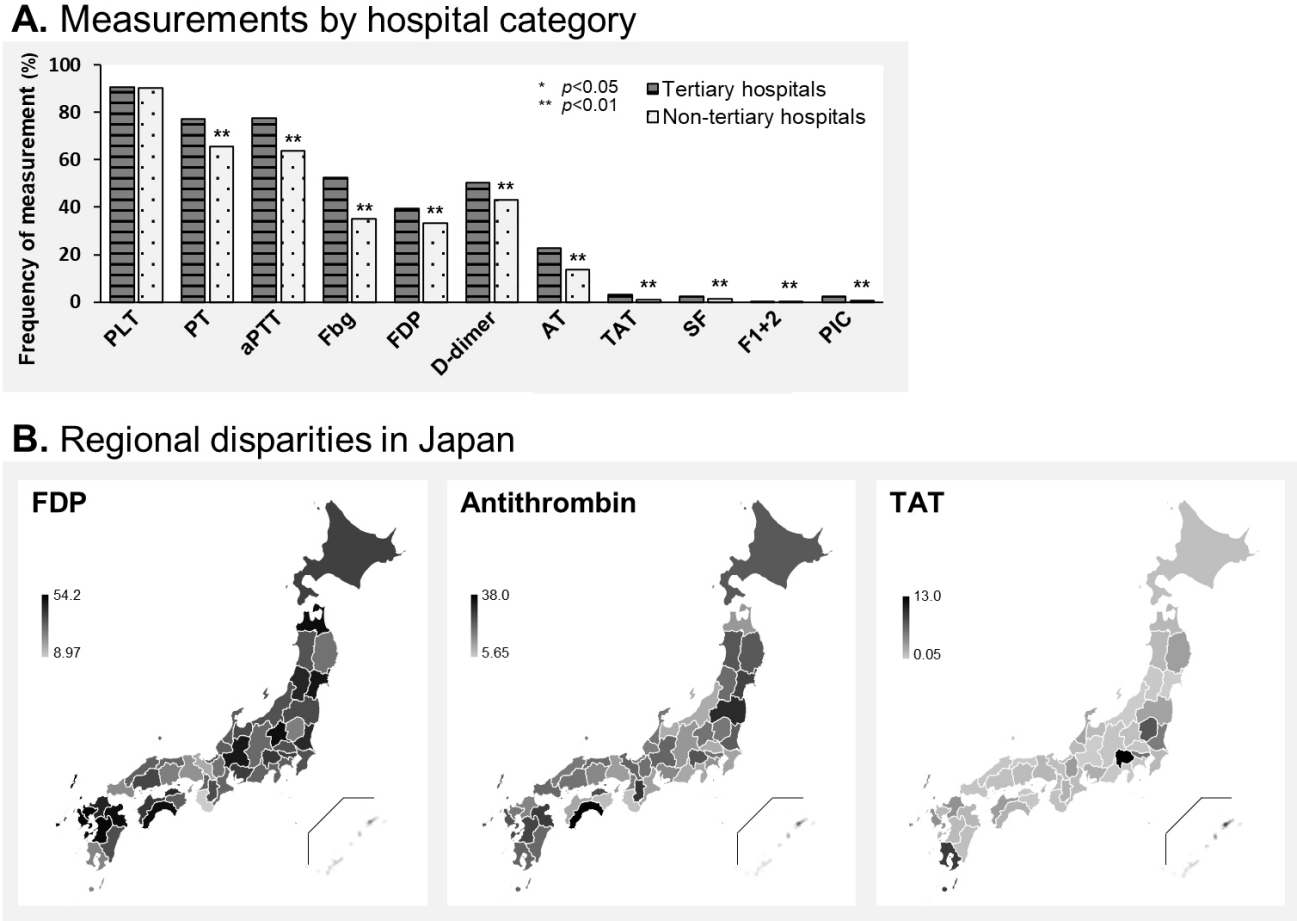
Measurement disparities according to hospital category and geographical location. (A) Frequency of measurement of hemostasis markers by category of tertiary hospital or not. Significance was determined by Pearson’s chi-squared test. (B) Heatmap showing the frequency of measurement of hemostasis markers by prefectures in Japan. PLT, platelet count; PT, prothrombin time; aPTT, activated partial thromboplastin time; Fbg, fibrinogen; FDP, fibrin degradation products; AT, antithrombin; TAT, thrombin-antithrombin complex; SF, soluble fibrin; F1+2, prothrombin fragment 1 + 2; PIC, plasmin-α2 plasmin inhibitor complex.

Measurement differences caused by hospital characteristic and geographical location are displayed in Figure 4. Regarding the hospital category, sepsis patients in tertiary and nontertiary emergency hospitals showed a similar frequency of measurement of global laboratory markers, whereas hemostasis markers (such as fibrinogen, D-dimer, and AT) were more frequently measured in tertiary rather than in nontertiary emergency hospitals (Figure 4A). The specific hemostasis molecular markers such as TAT, SF, F1+2, and PIC were seldom measured regardless of the hospital being tertiary or non-tertiary. Figure 4B shows a heatmap of frequency of measuring FDP, AT, and TAT by prefectures in Japan. FDP was measured more frequently in the Tohoku, Kanto, and Kyushu regions while less frequently in the Kinki and Chugoku. Similarly, AT was frequently measured in Tohoku and Kyushu. TAT was infrequently measured across Japan, except in a few prefectures. Disparities of measurement frequency around Japan are displayed for all three markers.

## Discussion

### Principal findings

In the present study, we first revealed the clinical practice of laboratory testing of hemostasis markers for sepsis management using a nationwide retrospective dataset of 153,474 adult hospitalized patients with sepsis in Japan. Regardless of the newly released JSTH DIC scoring system, hemostasis molecular markers such as TAT, SF, and F1+2 were seldom measured in clinical settings in Japan. The existing hemostasis markers such as fibrinogen or FDP that have been adopted by several DIC scoring systems were also not so frequently measured in Japan. Measurement of these clinically preferred hemostasis markers was impacted not only by the patient’s specific disease characteristics but also by hospital characteristic or geographical location.

### Clinical application of the findings

Now, several different clinical practice guidelines for DIC management have been developed by societies in Britain ^(18)^, Japan ^(19)^, and Italy ^(20)^, along with the harmonized Guidance by the International Society on Thrombosis and Haemostasis ^(21)^. Although some notable differences in the diagnosis of DIC and the utility of molecular markers exist between these guidelines, the general idea is consistent that “A combination of tests repeated over time in a patient with suspected DIC can be used to diagnose the disorder with reasonable certainty in most cases ^(21)^.” However, the clinical evidence on the current state of evaluating DIC or the use of hemostasis molecular markers in sepsis management has not been fully recognized. Umemura et al. reported that based on a large cohort of Japanese data including 2,663 adult sepsis patients, DIC screening was linked to a lower death rate in patients with sepsis ^(22)^. International surveys were conducted to evaluate current practice in DIC management for overall patients ^(23)^ and pediatric/neonatal patients ^(24)^. Both surveys demonstrated a largely heterogeneous approach of clinicians to the DIC diagnosis and clinical use of hemostasis laboratory tests. The findings consistently showed the limited use of diagnostic scores despite recommendations of the guidelines. The results of this investigation demonstrated that even in a high-risk sepsis cohort with a 30% mortality rate, suitable global hemostasis markers were not assessed upon admission and in the subsequent days. We surmise that there are several explanations for the poor adherence with the guidelines and the lack of appropriate marker evaluation. i) Physicians are unaware of the guideline suggestions; ii) even if they are aware of the guidelines, they do not feel the need to adhere to them (they think they can provide quality treatment without such blood testing); and iii) the costs of such laboratory testing are high. Further educational efforts from the relevant academic societies are warranted to improve the quality of DIC care in clinical practice.

The aforementioned four standards do not advise the routine use of hemostasis molecular markers such as TAT, SF, and F1+2. These molecular markers are known to reflect coagulation activation with greater sensitivity than global markers ^(25), (26), (27), (28)^ ^(29)^. The latest suggested JSTH DIC diagnostic criteria have applied these molecular markers and AT activity for the first time to improve both sensitivity and specificity for the diagnosis of DIC ^(12), (25)^. The findings of the present nationwide descriptive study showed the extremely low frequency of measurement of these hemostasis molecular markers in clinical settings in Japan, which suggests that the JSTH DIC diagnostic criteria are still not widely used at this time. More marketing campaigns are needed to communicate these standards to physicians at the clinical bedside.

### Limitations

We acknowledge several limitations in our study. First, we could not obtain the findings of any of the hemostasis tests. Furthermore, it is unclear from this study whether frequent hemostasis assessment is associated with changing treatment decisions and better patient outcomes. Additional prospective studies will be necessary to confirm the potential of adherence with the guidelines including DIC diagnosis and therapy to enhance patient outcomes. Second, diagnoses recorded in administrative claims databases are generally less accurate compared with those recorded in planned prospective studies. Although SOFA score data were included in this investigation, we did not thoroughly evaluate the baseline disease severity of each sepsis population. Finally, this study used Japanese data only, and thus, generalizability of the results to other countries may be limited. More research is required to understand the present practice regarding DIC diagnosis for sepsis using multinational datasets.

### Conclusions

The current clinical status of laboratory testing of hemostasis markers in Japan was first described based on the present nationwide retrospective observational study of 153,474 adult sepsis patients. Novel hemostasis molecular markers such as TAT, SF, and F1+2 were seldom measured in the clinical settings, and the current hemostasis markers such as fibrinogen or FDP that have been accepted by numerous DIC scoring systems were also not so frequently measured.

## Article Information

### Conflicts of Interest

Kazuma Yamakawa received research grants from Asahi Kasei Pharma and the Japan Blood Products Organization.

### Author Contributions

KY and HO contributed equally to this study. KY and HO developed and designed this study; contributed to collection, analysis, and interpretation of the data; and were responsible for writing, editing, and submission of the manuscript. RH and NU interpreted the data and contributed to editing of the manuscript. HM, KF, and HY had a significant influence on the interpretation of the findings and critical evaluation of the manuscript. All of the authors contributed to the acquisition of data and reviewed, discussed, and approved the final manuscript.

### Approval by Institutional Review Board (IRB)

The study was approved by the Institutional Review Board of the University of Tokyo [approval number: 3501-(5) (May 19, 2021)].

## Supplement

Supplementary Table 1
