# Supplementary material for: Current Clinical Practice of Laboratory Testing of the Hemostasis and Coagulation System in Patients with Sepsis: A Nationwide Observational Study in Japan
Source: JMA J. 2024 Feb 5;7(2):224–31. doi: 10.31662/jmaj.2023-0151 (PMC11074511; doi:10.31662/jmaj.2023-0151)
Supplement: Supplementary Table 1 [file 2433-3298-7-2-0224-s001.pdf]

**Supplementary Table 1** Definition of ICD-10 codes for types of sepsis	
Lung	A15 A16 A31 A37 B371 B440 B441 B450 J01 J02 J03 J04 J05 J06 J13 J14 J15 J16 J17 J18 J20 J21 J22 J440 J441 J47 J69 J85 J86
Abdomen	A020 A04 A05 A08 A09 A183 K35 K36 K37 K57 K61 K630 K631 K65 K750 K751 K800 K801 K803 K804 K808 K810 K830 K918
Urinary tract	N10 N11 N12 N151 N159 N160 N30 N34 N390 N41 N74 N75 T835 T836
Central nervous system	A17 A39 G00 G01 G02 G03 G04 G05 G06 G07 G08 G09
Skin/soft tissues	A33 A34 A35 A46 A48 B35 B36 L03 L04 L08 L726 L88
Cardiovascular	I30 I33 I80 T826 T827
Others	None of the above
